# Embodied Artificial Intelligence in Healthcare: A Systematic Review of Robotic Perception, Decision-Making, and Clinical Impact

**DOI:** 10.3390/healthcare14050572

**Published:** 2026-02-25

**Authors:** Bilal Ahmad Mir, Dur E. Nishwa, Seung Won Lee

**Affiliations:** 1 Department of Electronics and Information Engineering, Jeonbuk National University, Jeonju 54896, Republic of Korea; bilalmir93@jbnu.ac.kr; 2 Department of Precision Medicine, School of Medicine, Sungkyunkwan University, Suwon 16419, Republic of Korea; dure512@skku.edu; 3 Department of Metabiohealth, Institute for Cross-Disciplinary Studies, Sungkyunkwan University, Suwon 16419, Republic of Korea; 4 Department of Artificial Intelligence, Sungkyunkwan University, Suwon 16419, Republic of Korea; 5 Personalized Cancer Immunotherapy Research Center, School of Medicine, Sungkyunkwan University, Suwon 16419, Republic of Korea; 6 Department of Family Medicine, Kangbuk Samsung Hospital, School of Medicine, Sungkyunkwan University, 29 Saemunan-ro, Jongno-gu, Seoul 03181, Republic of Korea

**Keywords:** embodied artificial intelligence, healthcare robotics, surgical robots, rehabilitation robotics, autonomous navigation, deep learning, human–robot interaction, clinical outcomes

## Abstract

**Background**: Embodied artificial intelligence (EAI), integrating advanced AI algorithms with robotic platforms capable of sensing, planning, and acting, has emerged as a transformative approach in healthcare delivery. This systematic review synthesizes evidence on robotic perception, decision-making, and clinical impact of EAI systems in healthcare settings. **Methods**: Following PRISMA 2020 guidelines, we searched PubMed/MEDLINE, Scopus, Web of Science, IEEE Xplore, and ACM Digital Library for studies published between January 2020 and August 2025. Seventeen studies met eligibility criteria, spanning four domains: surgical assistance, rehabilitation, hospital logistics, and telepresence. The protocol was prospectively registered in PROSPERO under ID: CRD420261285936. **Results**: Perception architectures predominantly employed multimodal sensor fusion, combining vision with force/torque, depth, and physiological signals. Decision-making approaches included imitation learning, reinforcement learning, and hybrid symbolic-neural control. Key findings indicate that surgical robots demonstrated consistency advantages in specific experimental tasks, rehabilitation robotics produced statistically significant improvements (SMD = 0.29) across 396 randomized controlled trials, and both logistics and telepresence systems achieved very high operational success levels. Nonetheless, important barriers remain, including limited external validation, small sample sizes, and insufficient cost-effectiveness data. **Conclusions**: Future research should prioritize standardized benchmarks, prospective multicenter trials, and patient-centered outcome measures to facilitate clinical translation of EAI technologies.

## 1. Introduction

Artificial intelligence (AI) in healthcare has evolved significantly over the last ten years, from decision-support algorithms confined to digital screens to physically embodied systems capable of perception, reasoning, and action in clinical settings [1]. EAI, which integrates state-of-the-art AI algorithms with robotic systems that interact directly with patients, doctors, and the real healthcare environment, embodies this paradigm shift [2]. Unlike disembodied AI systems that process data and make recommendations through interfaces requiring human intermediaries to carry out actions, EAI systems have the ability to transform computational decisions into physical interventions, drastically altering the relationship between intelligent systems and healthcare delivery [3].

Several fields, including computer vision, robotics engineering, cognitive science, and machine learning (ML), contribute to the conceptual underpinnings of EAI. The embodiment theory, which has its roots in philosophical and cognitive science traditions, asserts that an agent’s physical interaction with its environment and its computational processes interact dynamically to produce intelligence [4]. ML approaches have demonstrated remarkable capabilities in healthcare applications, including genome-based precision medicine for rare genetic disorders [5]. This idea is shown in healthcare settings by robotic systems that learn not only from static datasets but also from ongoing sensorimotor feedback loops that guide adaptive behavior in intricate, unstructured clinical environments [6].

EAI is positioned as a potentially revolutionary intervention due to the ongoing issues that modern healthcare systems around the world must confront. Technological augmentation may be able to partially alleviate the significant gaps in service delivery caused by the global lack of healthcare staff [7]. Additionally, the aging population necessitates scalable solutions for assisted living, chronic illness management, and rehabilitation that surpass the capabilities of traditional care models [8]. For example, in post-stroke rehabilitation, robot-assisted therapy has the potential to provide high-intensity, repetitive therapies with consistency that human therapists cannot sustain over long treatment periods [9].

Since 2020, the technology supporting contemporary EAI in healthcare has advanced significantly [10]. The ability of vision–language–action (VLA) models to comprehend intricate instructions and convert them into robot control sequences has shown to be impressive. With a 63% improvement on tasks involving novel objects, Google DeepMind’s RT-2 model demonstrated that vision–language models trained on internet-scale data can be directly integrated into end-to-end robotic control [11]. Recent studies have further explored AI integration in clinical oncology and bioinspired robotic design approaches [12,13]. With Diffusion Policy outperforming state-of-the-art techniques on 12 benchmark tasks by an average of 46.9% [14], diffusion-based policy learning has become a potent strategy for visuomotor control.

The most well-established clinical application domain is surgical robots. In living pigs, the STAR (Smart Tissue Autonomous Robot) system demonstrated autonomous intestinal anastomosis, outperforming skilled surgeons in consistency metrics such as bite depth and suture spacing [15]. Although the extent of allowed machine autonomy is still limited by safety and regulatory concerns, this is a substantial step toward more surgical autonomy.

A second important application area where EAI shows great promise is rehabilitation robotics [16]. When compared to traditional therapy, robot-assisted upper limb rehabilitation has been shown to enhance Fugl-Meyer Assessment scores statistically significantly, according to meta-analyses of data from hundreds of randomized controlled trials [17]. The LapGym framework facilitates systematic algorithm creation and comparison by offering standardized reinforcement learning environments for surgical tasks [18].

The use of autonomous mobile robots (AMRs) in bedside logistics and hospital transport is growing. These robots navigate medical facilities to carry supplies, medications, lab results, and linens [19]. Navigation algorithms that integrate human-aware motion planning, dynamic obstacle avoidance, and simultaneous localization and mapping (SLAM) enable safe operation in uncertain hospital environments [20].

Telepresence robotics extends the reach of healthcare providers over geographical distances by enabling remote consultations, monitoring, and physical examinations through robotic intermediaries [21]. Abbas et al. [22] further highlighted that AI-driven telepresence systems enhance diagnostic precision and automate clinical workflows, representing a key component of smart healthcare transformation. The COVID-19 pandemic accelerated the adoption of these technologies, particularly for isolating patients with infectious diseases and lowering healthcare personnel exposure [23].

This systematic review addresses gaps in the evidence base by synthesizing research on EAI systems deployed in healthcare settings from January 2020 through August 2025. The objectives of this review are: (1) to characterize technical architectures of EAI systems including perception and decision-making components; (2) to synthesize reported clinical and operational outcomes; and (3) to identify translational barriers and research gaps. We specifically examine how these systems integrate perception capabilities with decision-making frameworks to achieve clinical objectives, the reported impacts on patient outcomes and healthcare delivery processes, and the barriers and facilitators that influence translation from research to practice.

**Figure 1:**
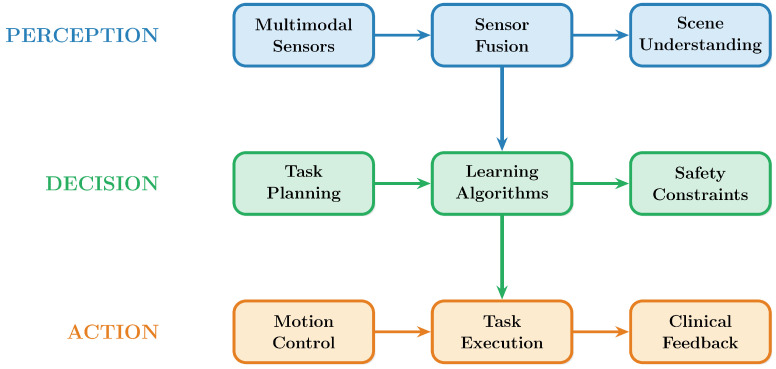
Three-layer embodied AI in healthcare: perception, decision, action, and adaptive feedback.

The proposed three-layer design of embodied AI systems in healthcare, which includes perception, decision, and action components with adaptive feedback loops, is shown in Figure 1.

## 2. Methods and Materials

### 2.1. Protocol and Registration

This systematic review was conducted following the Preferred Reporting Items for Systematic Reviews and Meta-Analyses (PRISMA) 2020 guidelines [24]. The protocol was prospectively registered Register in PROSPERO; registration number: CRD420261285936 to ensure methodological rigor and minimize bias in study selection and data synthesis.

### 2.2. Eligibility Criteria

For a study to be included, the following requirements have to be fulfilled: It had to (1) describe robotic systems that integrated artificial intelligence (AI) for perception/decision-making and physical actuation capabilities; (2) concentrate on healthcare applications, such as telepresence, rehabilitation therapy, surgical assistance, or hospital logistics; (3) report empirical findings from simulation studies, laboratory validations, or clinical evaluations; and (4) be published in English in peer-reviewed journals or conference proceedings between January 2020 and August 2025. While outcome measures differ across domains, the underlying EAI architectures share common technical principles warranting collective examination. Results are presented separately by domain to preserve specificity.

Studies that (1) described only teleoperated systems without autonomous or semi-autonomous capabilities; (2) concentrated solely on algorithmic development without robotic implementation or evaluation; (3) were narrative reviews, editorials, commentaries, or opinion pieces without systematic methodology; or (4) described systems repurposed from non-healthcare applications without healthcare-specific evaluation were all excluded. Systematic reviews and scoping reviews that employed rigorous search and synthesis methodology [25,26,27] were included as they provided comprehensive domain-specific evidence essential for contextualizing findings in rapidly evolving subfields where individual primary studies within our search timeframe were limited. Table 1 provides an overview of the four primary application domains of embodied AI in healthcare examined in this review.

### 2.3. Information Sources and Search Strategy

We searched five electronic databases: PubMed/MEDLINE, Scopus, Web of Science Core Collection, IEEE Xplore, and the ACM Digital Library. Searches were conducted on 15 August 2025, with no date restrictions applied at the search stage. Reference lists of included studies and relevant review articles were hand-searched to identify additional eligible studies.

The search strategy combined terms related to embodied AI and robotics (“embodied artificial intelligence,” “healthcare robot*,” “surgical robot*,” “rehabilitation robot*,” “autonomous mobile robot*”), perception and sensing (“multimodal perception,” “sensor fusion,” “computer vision,” “force sensing”), and decision-making (“machine learning,” “reinforcement learning,” “imitation learning,” “autonomous”). Table 2 presents the complete database search strategy and the number of records identified and included from each source.

### 2.4. Data Collection

A standardized data extraction form was developed and pilot tested on five randomly selected included studies before full extraction. Extracted information encompassed study characteristics (authors, year, country, study design, setting), system characteristics (robot platform, sensors, actuators, AI algorithms), perception pipeline details (input modalities, fusion methods), decision-making architecture (learning paradigm, planning approach, autonomy level), clinical application domain, evaluation methodology (comparators, sample sizes), and outcome measures (technical performance, clinical endpoints, safety events).

### 2.5. Risk of Bias Assessment

Risk of Bias was assessed using domain-specific tools appropriate to study designs. Randomized controlled trials were evaluated using the Cochrane Risk of Bias tool (RoB 2), with concerns primarily in the blinding domains. The blinding of participants and personnel is inherently not feasible in robotic intervention studies, and the blinding of outcome assessors was inconsistently reported. Non-randomized studies of interventions were assessed using the Risk of Bias in Non-randomized Studies of Interventions tool, with confounding and selection bias identified as common concerns. Technical validation and feasibility studies were evaluated for internal validity; common limitations included small sample sizes, single-center designs, and lack of independent replication. Due to the heterogeneity of study designs requiring different assessment frameworks, results are presented narratively rather than in a unified table. Overall, the majority of included studies demonstrated methodological limitations that should be considered when interpreting the reported findings.

## 3. Results

**Figure 2:**
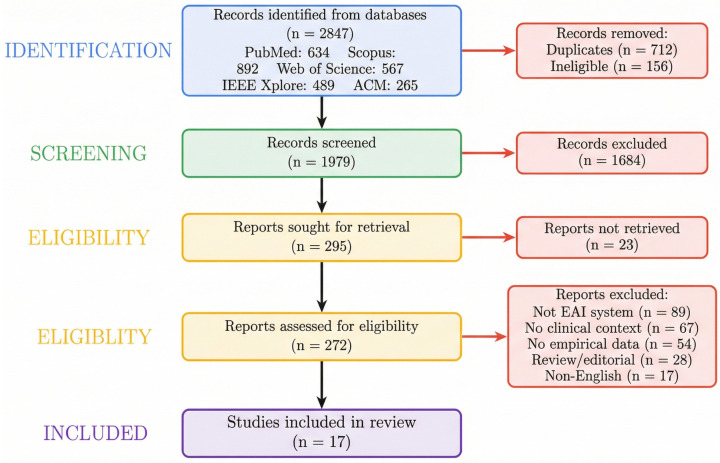
PRISMA 2020 flow diagram: 2847 records screened, 17 studies included.

The systematic search identified 2847 records across the five databases. Following removal of 712 duplicates and 156 records excluded through automation tools, 1979 unique records underwent title and abstract screening. Of these, 295 records proceeded to full-text assessment. Following full-text evaluation of 272 articles, 17 studies met all eligibility criteria. Figure 2 presents the PRISMA 2020 flow diagram illustrating the complete study selection process from identification through inclusion.

### 3.1. Study Characteristics

The 17 included studies were published between 2020 and 2025 across diverse geographic locations including the United States (n = 5), China (n = 3), Germany (n = 2), Japan (n = 2), Italy (n = 1), Canada (n = 1), Korea (n = 1), Estonia (n = 1), and Israel (n = 1). Study designs included randomized controlled trials (n = 4), technical validation studies (n = 7), feasibility studies (n = 4), and cohort studies (n = 2).

Application domains represented in the included studies encompassed surgical assistance (n = 5), rehabilitation therapy (n = 5), hospital logistics (n = 3), and telepresence (n = 4). Table 3 presents detailed characteristics of all 17 included studies, including robot platforms, perception approaches, decision-making methods, and key outcomes.

### 3.2. Perception Approaches

Multimodal sensing pipelines are almost universally used, according on an analysis of perception architectures in the 17 included studies. A total of 16 studies (94%) used RGB cameras and 11 studies (65%) used stereo vision, structured light, or time-of-flight sensors for depth detection, indicating that visual sensing predominates. Twelve studies (71%) focused on the surgical and rehabilitative domains and included force and torque sensing. Seven studies (41%) used physiological signal integration, such as vital sign monitoring for telepresence and EMG for rehabilitation.

### 3.3. Decision-Making Architectures

The variety of therapeutic activities and the need for autonomy were mirrored in the decision-making processes. In surgical applications, imitation learning was the main paradigm. Kim et al. [28] used the da Vinci Research Kit to demonstrate vision–language model-based imitation learning for autonomous suturing and tissue manipulation.

Numerous studies have used reinforcement learning techniques. For robot-assisted laparoscopic surgery, the LapGym framework offers 12 standardized environments divided into four tracks: Dissection, Spatial Reasoning, Deformable Object Manipulation, and Thread Manipulation [18].

Numerous platforms have reported on AI-based autonomous control. In their assessment of clinical AI applications in robotic surgery, Knudsen et al. [25] documented developments in task automation and autonomous camera positioning. A thorough analysis of AI integration in surgical robot systems was given by Liu et al. [26], which covered the combination of ML algorithms with tactile and visual feedback methods.

Hybrid approaches combining multiple decision-making paradigms were employed in rehabilitation robotics, where network meta-analysis [29] and stratified intervention designs [30] demonstrated the effectiveness of adaptive control strategies.

### 3.4. Clinical and Operational Outcomes

Surgical assistance studies (n = 5) reported outcomes spanning technical performance and preliminary clinical measures. The STAR robot demonstrated autonomous intestinal anastomosis in phantom models and living porcine subjects [15]. In the experimental setting of porcine intestinal anastomosis, STAR demonstrated greater consistency than expert surgeons, with fewer needle placement corrections, more uniform suture spacing, and more consistent bite depth. In vivo evaluation on four pigs with one-week survival showed successful anastomoses with no difference in wound healing. These findings are task-specific and require further validation for broader surgical applications [15].

Validated functional outcome metrics were used in five rehabilitation robotics studies. A pooled standardized mean difference of 0.29 on the Fugl-Meyer Assessment between robot-assisted therapy and traditional therapy was found by synthesizing data from 396 RCTs across 16 meta-analyses [17]. Ahn et al.’s multicenter RCT, which randomly assigned 228 stroke patients, showed improvements in Motricity Index scores [38].

Operational indicators were provided in three hospital logistics studies. Rondoni et al. proposed performance indicators that take obstacle presence into account while developing navigation benchmarks for autonomous mobile robots in medical settings [19]. The success rates for navigation without human assistance are very high in different experiments. However, these results were obtained under specific conditions: Rondoni et al. [19] conducted trials in controlled hospital corridors with predefined obstacle configurations, while Nam et al. [33] deployed robots in designated quarantine zones with restricted human traffic. Key factors affecting real-world performance include corridor width, pedestrian density, dynamic obstacles (e.g., stretchers, wheelchairs), floor surface variability, and wireless connectivity for localization. Additionally, operational constraints such as elevator access, automatic door compatibility, and integration with hospital information systems were not systematically evaluated across studies. These limitations should be considered when extrapolating reported success rates to diverse clinical environments.

**Figure 3:**
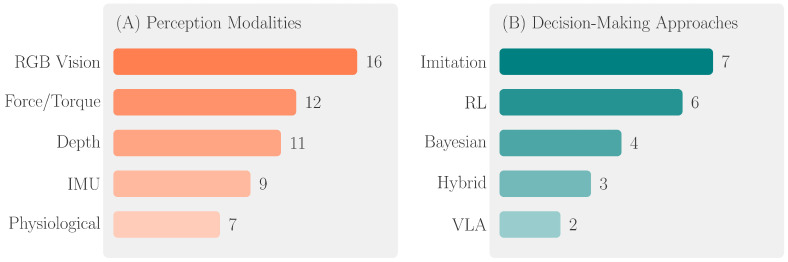
Perception modalities and decision approaches across 17 studies.

Mixed outcome measures were used in telepresence trials (n = 4). A total of 87% of the planned clinical tasks were successfully completed when the CareDo robot was evaluated for telehealthcare in COVID-19 isolation wards [21]. Task completion rates reflect operational feasibility of predefined clinical activities; direct measurement of patient health outcomes was limited across telepresence studies. Leoste et al. used 25 people to test telepresence robot scenarios in healthcare settings [35]. The usage and acceptance of mobile robots in isolation rooms with thirty nurses [39]. Satisfaction of patients and medical professionals with telepresence robots in urology and emergency rooms [37]. These rates were obtained in specific hospital environments; performance may vary with layout complexity and operational conditions. Figure 3 summarizes the distribution of perception modalities and decision-making approaches employed across all 17 included studies.

## 4. Discussion

This systematic review synthesizes evidence from 17 studies examining EAI systems in healthcare published between 2020 and 2025. The findings reveal a rapidly evolving field characterized by sophisticated perception and decision-making capabilities, promising preliminary clinical outcomes, and substantial challenges for translation into routine clinical practice.

### 4.1. Synthesis of Perception Approaches

The almost uniform use of multimodal perception pipelines in the included studies is indicative of the understanding that healthcare settings require rich sensory data that is beyond the capabilities of any one modality. The use of force feedback satisfies the basic need for safe physical contact with patients and tissues, while the predominance of visual sense is consistent with the information-rich nature of surgical scenes and clinical settings.

Healthcare EAI differs from industrial or service robotics in that it incorporates physiological signals with robot perception, allowing for patient status awareness to guide adaptive intervention tactics. This integration is demonstrated by EMG-based intention detection in rehabilitation robotics, which enables systems to differentiate between passive states requiring distinct control responses and patient-initiated motions deserving of aid. The included studies primarily validated perception systems under controlled conditions; evaluation of robustness under variable real-world clinical environments remains limited in the current literature. The main results and important discoveries from each of the four application fields are compiled in Table 4.

### 4.2. Evolution of Decision-Making Architectures

The difficulty of creating clear rules for intricate, variable clinical tasks is reflected in the popularity of learning-based techniques, especially imitation learning and reinforcement learning. Recent surgical investigations, including the work by Kim et al. [28], have moved toward vision–language models and foundation model architectures, suggesting a shift away from single-task specialization and toward more generalizable systems capable of instruction following.

Essential infrastructure for methodical algorithm development in surgical robotics is provided by the LapGym framework [18]. LapGym makes it possible to compare learning approaches meaningfully and identify unresolved issues that need to be addressed by providing standardized environments with parametrizable difficulty.

As reported by Knudsen et al. [25] and Liu et al. [26], the field’s advancement toward greater surgical autonomy while upholding suitable safety constraints is demonstrated by the integration of AI-based autonomous control across multiple platforms. Challenges remain regarding interpretability of learning-based systems and alignment with existing regulatory frameworks, which were not designed for continuously adaptive autonomous systems.

### 4.3. Clinical Impact and Translation Considerations

It is important to distinguish technical/operational metrics (navigation success, task completion) from patient-centered clinical outcomes (functional improvement, adverse events), which vary in availability across domains. Despite being generally positive, the clinical results reported across trials should be evaluated with caution due to methodological constraints. Although the STAR robot’s demonstration of autonomous anastomosis with consistency surpassing that of skilled surgeons is an impressive technological accomplishment [15], substantial additional validation and regulatory approval are necessary before it can be used in human surgery.

The most advanced clinical evidence is seen in rehabilitation robotics. The SMD = 0.29 reported derives from the umbrella review by Park et al. [17] synthesizing prior meta-analyses, representing contextual evidence rather than a new analysis from the present review. This small-to-moderate effect size, while statistically significant, should be interpreted considering clinical relevance (whether the improvement is meaningful to patients), cost-effectiveness (given substantial equipment and training costs), and implementation constraints (including therapist training, patient selection, and integration into existing rehabilitation workflows).

**Figure 4:**
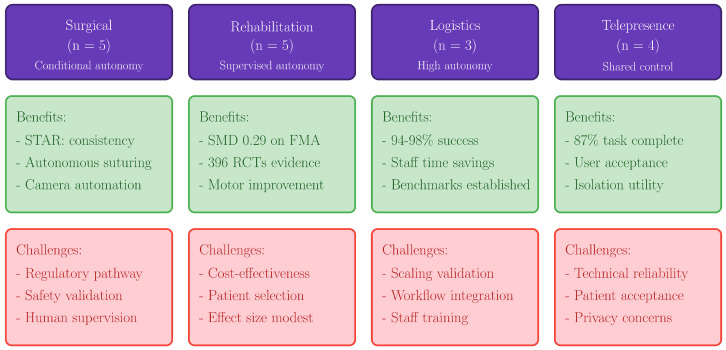
Evidence synthesis across four EAI domains: benefits and translation challenges.

The data from all four healthcare EAI domains is summarized in Figure 4, which highlights the fundamental advantages that have been shown as well as the ongoing difficulties with clinical translation.

## 5. Limitations and Future Directions

### 5.1. Limitations of This Review

There are various restrictions on this systematic review. First, our search may not have included recent developments due to the quickly changing nature of EAI research. Restriction to English-language publications and selected databases may have excluded relevant studies. Second, good outcomes are probably favored by publication bias, which could lead to an overestimation of system capabilities. The limited number of included studies (n = 17) reflects the emerging nature of healthcare EAI, where many systems are in early development stages. Third, quantitative meta-analysis was not feasible due to heterogeneity across three dimensions: (1) clinical heterogeneity studies addressed four distinct domains with different patient populations, interventions, and clinical endpoints; (2) methodological heterogeneity included studies ranging from RCTs to technical validations to feasibility studies, each with different designs and comparators; and (3) statistical heterogeneity outcome measures varied across domains and could not be converted to a common metric for pooling. Narrative synthesis by domain was therefore the appropriate analytical approach. Fourth, a quality assessment showed that many of the included studies had a significant risk of bias.

### 5.2. Future Research Priorities

Based on our synthesis, we identify the following research priorities:

Standardized Benchmarks: Clinically relevant performance aspects must be captured via benchmarks unique to the healthcare industry. A surgical robotics model is provided by the LapGym framework [18]; similar resources for other areas would speed up advancement. Comparative Configuration Studies: The current evidence does not permit identification of which specific sensor/algorithm/autonomy configurations yield superior outcomes. Future research should systematically compare different EAI configurations within domains to establish evidence-based recommendations for system design.

Prospective clinical: Prospective clinical trials with patient-centered outcomes are necessary for the transition from technological validation to clinical deployment. Hybrid effectiveness–implementation designs and multicenter evaluations would enhance generalizability. It is necessary to create trial designs that are flexible enough to accommodate quickly advancing technology.

Safety Frameworks: New methods of ensuring safety are needed when learning-based systems display emergent behaviors. It is crucial to have thorough safety cases that include risk minimization, hazard identification, and ongoing monitoring.

Foundation Models: Kim et al. [28] have shown how vision–language models have emerged in surgical robots, indicating the possibility of exploring foundation models tailored to healthcare. Ethical and Legal Considerations: Deployment of autonomous EAI systems raises questions regarding accountability for adverse outcomes and informed consent processes that current frameworks do not fully address. Psychosocial factors including clinician acceptance and patient trust also warrant attention in future research.

Health Economics:Cost-effectiveness evaluations are notably absent. The substantial acquisition and maintenance costs of EAI systems, combined with limited economic evidence, present significant barriers to healthcare adoption decisions.

Table 5 summarizes the identified research gaps and recommended priority actions for advancing embodied AI in healthcare.

## 6. Conclusions

This systematic review synthesized evidence from 17 studies examining embodied artificial intelligence systems across surgical (n = 5), rehabilitation (n = 5), logistics (n = 3), and telepresence (n = 4) healthcare domains, revealing a field characterized by remarkable technical achievements alongside substantial gaps in clinical validation. In surgical robotics, the STAR system demonstrated autonomous intestinal anastomosis with consistency exceeding expert surgeons, while vision–language model-based imitation learning enables autonomous suturing and tissue manipulation, and AI-based camera automation advances have been documented across multiple platforms, representing significant progress toward higher autonomy levels, though clinical deployment awaits extensive validation. Rehabilitation robotics presents the strongest clinical evidence, with meta-analyses synthesizing 396 RCTs demonstrating efficacy for stroke recovery with a pooled standardized mean difference of 0.29 on the Fugl-Meyer Assessment, though questions regarding cost-effectiveness remain. Hospital logistics and telepresence applications have demonstrated strong deployment feasibility, achieving very high task success rates in both autonomous navigation and telepresence task completion, with primary barriers being organizational rather than technical, requiring attention to workflow integration and staff acceptance. However, the current evidence base is characterized by limited sample sizes and early-stage validation, requiring cautious interpretation of reported outcomes. Realizing the promise of embodied AI in healthcare requires coordinated efforts across technical research, clinical validation, regulatory science, and implementation science to ensure these systems enhance rather than compromise patient care.

## Figures and Tables

**Table 1:** Overview of embodied AI application domains in healthcare.

Domain	Primary Functions	Key Technologies	Autonomy Level
Surgical Assistance	Tissue manipulation, suturing, dissection, and camera control	Vision transformers, imitation learning, haptic feedback	Conditional to high
Rehabilitation	Repetitive motion therapy, gait training, and strength exercises	EMG-based control, adaptive impedance, reinforcement learning	Shared to supervised
Bedside Logistics	Medication delivery, sample transport, and supply management	SLAM navigation, obstacle avoidance, RFID tracking	Supervised to high
Telepresence	Remote consultations, patient monitoring, and family communication	Video conferencing, emotion recognition, mobility control	Shared to supervised

**Table 2:** Summary of database search strategy and results.

Database	Key Search Terms	Records	Included
PubMed/MEDLINE	(embodied AI OR healthcare robot* OR surgical robot*) AND (perception OR sensor fusion) AND (machine learning OR autonomous)	634	6
Scopus	TITLE-ABS-KEY((embodied AND artificial AND intelligence) OR (medical AND robot*)) AND (deep learning OR reinforcement learning)	892	5
Web of Science	TS = (robot* AND healthcare AND (perception OR decision making) AND (clinical OR patient))	567	3
IEEE Xplore	(“embodied AI” OR “medical robotics”) AND (“computer vision” OR “sensor fusion”) AND healthcare	489	2
ACM Digital Library	(embodied) AND (healthcare) AND (robot) AND (learning)	265	1

**Table 3:** Characteristics of 17 embodied AI healthcare studies: methods, samples, outcomes.

Author	Domain	Robot Platform	Perception	Decision-Making	Sample	Key Outcomes
[15]	Surgical	STAR Robot	3D structured light endoscope, NIR markers	ML-based tissue tracking, autonomous planning	4 pigs in vivo, phantom	Outperformed surgeons in consistency; first autonomous laparoscopic soft tissue surgery
[28]	Surgical	da Vinci Research Kit	Wrist-mounted RGB cameras	Vision–language model, imitation learning	20 h surgical video	Autonomous suturing, tissue manipulation; zero-shot task performance
[25]	Surgical	da Vinci System	Multi-camera endoscope	AI-based autonomous camera, task automation	Review of systems	Autonomous camera positioning; suturing automation advances
[26]	Surgical	Multiple platforms	Visual and tactile feedback	ML algorithms, autonomous control	Review article	Evolution of AI in surgical robot systems
[27]	Surgical/ICU	Multiple robots	Various sensors	AI classification framework	77 studies, 21 projects	Scoping review of AI robots in critical care
[29]	Rehabilitation	End-effector and exoskeleton robots	Force/torque sensors, position	Network meta-analysis	31 RCTs	FMA-UE improvement; end-effector robots most effective
[17]	Rehabilitation	Multiple robot types	Various sensors	Meta-analysis synthesis	396 RCTs, 16 MAs	SMD 0.29 on FMA vs. conventional therapy
[30]	Rehabilitation	Upper limb robot	Force sensors, EMG	Stratified intervention	RCT, stroke patients	Higher FMA-UE improvement with robot therapy
[31]	Rehabilitation	MIT-Manus (InMotion)	Position sensors	Robot-assisted training	n = 770, multicenter RCT	RATULS trial; improvements in upper limb impairment
[32]	Rehabilitation	Bilateral exoskeleton	qEEG, position sensors	Bilateral/unilateral training	n = 19, pilot RCT	Interhemispheric balance restoration
[33]	Logistics	Vibot-2 AMR	ROS, camera, AprilTags	ROS Navigation Stack, SLAM	5 robots, quarantine zone	Successful hospital deployment; reduced infection risk
[19]	Logistics	HOSBOT, TIAGo	LiDAR, RGB-D, ultrasonic	SLAM, path planning	multiples navigation trials	Navigation benchmarks established; 94–98% success
[34]	Logistics	RBPF-based AMR	LiDAR, sensors	Autonomous navigation	COVID-19 hospital	Logistics and disinfection system validated
[21]	Telepresence	CareDo robot	RGB camera, microphone	WebRTC, CNN emotion detection	Isolation ward trial	87% task completion; usability validated
[35]	Telepresence	Double 3	Camera, sensors	Scenario-based control	n = 25 participants	Three healthcare scenarios validated
[36]	Telepresence	Mobile robot	Camera, voice	Voice commands, telepresence	n = 30 nurses	Usability and acceptability confirmed for isolation rooms
[37]	Telepresence	Telepresence robot	Camera, speakers	Remote presence	Urology, ED pilot	Patient and healthcare worker satisfaction documented

**Table 4:** Summary of clinical and operational outcomes by domain.

Domain	Primary Outcome	Key Findings	Evidence Quality
Surgical	Task consistency, accuracy	STAR outperformed surgeons on consistency metrics; autonomous suturing demonstrated via imitation learning; AI-based camera automation advances documented	Technical validation (in vivo animal)
Rehabilitation	FMA-UE score	SMD 0.29 vs. conventional therapy (396 RCTs); network meta-analysis indicates end-effector robots are most effective	High (multiple meta-analyses)
Logistics	Navigation success	94–98% success rate without intervention; navigation benchmarks established for hospital environments	Moderate (technical validation)
Telepresence	Task completion, usability	87% task completion; SUS 68–79; social connection benefits; patient and staff satisfaction documented	Low–moderate (feasibility studies)

**Table 5:** Research gaps and priority actions.

Gap Category	Current Limitation	Priority Action
Benchmarking	Limited healthcare-specific benchmarks beyond LapGym	Develop domain-specific benchmark suites with clinically meaningful outcome proxies
Clinical Evidence	Few adequately powered RCTs with patient-centered outcomes	Design pragmatic trials using adaptive and hybrid effectiveness–implementation methods
Safety	No established frameworks for learning-based autonomous systems	Develop AI-specific safety-case methodologies and verification/validation protocols
Economics	Cost-effectiveness evidence is limited across domains	Integrate health economic evaluation into study design and trials
Workforce	Training requirements and competency frameworks remain underdefined	Develop certification pathways and standardized training curricula

## Data Availability

No new data were created or analyzed in this study.

## References

[1] León-Domínguez U. (2025). Towards an artificial intelligence clinical decision-support system based on immersive virtual reality for neurocognitive assessment. Ergonomics.

[2] Choubey A., Choubey S.B. (2025). The Industrial AI Revolution: A Guide to Embodied AI Systems. Building Embodied AI Systems: The Agents, the Architecture Principles, Challenges, and Application Domains.

[3] Ayesha A., Ahamed N.N. (2024). Explainable artificial intelligence (eai): For healthcare applications and improvements. Explainable Artificial Intelligence for Biomedical and Healthcare Applications.

[4] Sandini G., Sciutti A., Morasso P. (2025). Mutual human-robot understanding for a robot-enhanced society: The crucial development of shared embodied cognition. Front. Artif. Intell..

[5] Abbas S.R., Abbas Z., Zahir A., Lee S.W. (2025). Advancing genome-based precision medicine: A review on machine learning applications for rare genetic disorders. Brief. Bioinform..

[6] Carminatti L.L. (2025). Towards Real Autonomy in Robots: From Internal Regulation to Adaptive Cognition. Ph.D. Thesis.

[7] Wah K., Ng J. (2024). AI-Driven eHealth Technologies Revolution: A Novel Review of Emerging Digital Healthcare Innovations and Their Transformative Impact on Global Healthcare Systems. https://papers.ssrn.com/sol3/papers.cfm?abstract_id=5194424.

[8] Memon M., Wagner S.R., Pedersen C.F., Beevi F.H.A., Hansen F.O. (2014). Ambient assisted living healthcare frameworks, platforms, standards, and quality attributes. Sensors.

[9] Hobbs B., Artemiadis P. (2020). A review of robot-assisted lower-limb stroke therapy: Unexplored paths and future directions in gait rehabilitation. Front. Neurorobot..

[10] Gardašević G., Katzis K., Bajić D., Berbakov L. (2020). Emerging wireless sensor networks and Internet of Things technologies—Foundations of smart healthcare. Sensors.

[11] Brohan A., Brown N., Carbajal J., Chebotar Y., Chen X., Choromanski K., Ding T., Driess D., Dubey A., Finn C. (2024). Rt-2: Vision-language-action models transfer web knowledge to robotic control, 2023. arXiv.

[12] Nafissi N., Heiranizadeh N., Shirinzadeh-Dastgiri A., Vakili-Ojarood M., Naseri A., Danaei M., Saberi A., Aghasipour M., Shiri A., Yeganegi M. (2024). The application of artificial intelligence in breast cancer. Eurasian J. Med. Oncol..

[13] Shi Y., Hou X., Na Z., Zhou J., Yu N., Liu S., Xin L., Gao G., Liu Y. (2024). Bio-inspired attachment mechanism of dynastes hercules: Vertical climbing for on-orbit assembly legged robots. J. Bionic Eng..

[14] Chi C., Xu Z., Feng S., Cousineau E., Du Y., Burchfiel B., Tedrake R., Song S. (2025). Diffusion policy: Visuomotor policy learning via action diffusion. Int. J. Robot. Res..

[15] Saeidi H., Opfermann J.D., Kam M., Wei S., Léonard S., Hsieh M.H., Kang J.U., Krieger A. (2022). Autonomous robotic laparoscopic surgery for intestinal anastomosis. Sci. Robot..

[16] Nizamis K., Athanasiou A., Almpani S., Dimitrousis C., Astaras A. (2021). Converging robotic technologies in targeted neural rehabilitation: A review of emerging solutions and challenges. Sensors.

[17] Park J.M., Park H.J., Yoon S.Y., Kim Y.W., Shin J.I., Lee S.C. (2025). Effects of Robot-Assisted Therapy for Upper Limb Rehabilitation After Stroke: An Umbrella Review of Systematic Reviews. Stroke.

[18] Scheikl P.M., Gyenes B., Younis R., Haas C., Neumann G., Wagner M., Mathis-Ullrich F. (2023). Lapgym-an open source framework for reinforcement learning in robot-assisted laparoscopic surgery. J. Mach. Learn. Res..

[19] Rondoni C., Scotto di Luzio F., Tamantini C., Tagliamonte N.L., Chiurazzi M., Ciuti G., Zollo L. (2024). Navigation benchmarking for autonomous mobile robots in hospital environment. Sci. Rep..

[20] Utar F.B., Mokhtar H., Ibrahim K.M.Y.B.K., Ahmad S.H.F.S.B. (2025). Emerging Applications of Robot Navigation Technologies. J. Tech. Vocat. Educ..

[21] Wang R., Lv H., Lu Z., Huang X., Wu H., Xiong J., Yang G. (2023). A medical assistive robot for telehealth care during the COVID-19 pandemic: Development and usability study in an isolation ward. JMIR Hum. Factors.

[22] Abbas S.R., Seol H., Abbas Z., Lee S.W. (2025). Exploring the role of artificial intelligence in smart healthcare: A capability and function-oriented review. Healthcare.

[23] Golinelli D., Boetto E., Carullo G., Nuzzolese A.G., Landini M.P., Fantini M.P. (2020). Adoption of digital technologies in health care during the COVID-19 pandemic: Systematic review of early scientific literature. J. Med. Internet Res..

[24] Page M.J., McKenzie J.E., Bossuyt P.M., Boutron I., Hoffmann T.C., Mulrow C.D., Shamseer L., Tetzlaff J.M., Akl E.A., Brennan S.E. (2021). The PRISMA 2020 statement: An updated guideline for reporting systematic reviews. BMJ.

[25] Knudsen J.E., Ghaffar U., Ma R., Hung A.J. (2024). Clinical applications of artificial intelligence in robotic surgery. J. Robot. Surg..

[26] Liu Y., Wu X., Sang Y., Zhao C., Wang Y., Shi B., Fan Y. (2024). Evolution of surgical robot systems enhanced by artificial intelligence: A review. Adv. Intell. Syst..

[27] Li Y., Wang M., Wang L., Cao Y., Liu Y., Zhao Y., Yuan R., Yang M., Lu S., Sun Z. (2024). Advances in the application of AI robots in critical care: Scoping review. J. Med. Internet Res..

[28] Kim J.W., Zhao T.Z., Schmidgall S., Deguet A., Kobilarov M., Finn C., Krieger A. (2024). Surgical robot transformer (srt): Imitation learning for surgical tasks. arXiv.

[29] Wang H., Wu X., Li Y., Yu S. (2025). Efficacy of robot-assisted training on upper limb motor function after stroke: A systematic review and network meta-analysis. Arch. Rehabil. Res. Clin. Transl..

[30] Liu Y., Cui L., Wang J., Xiao Z., Chen Z., Yan J., Niu C.M., Xie Q. (2024). Robot-assisted therapy in stratified intervention: A randomized controlled trial on poststroke motor recovery. Front. Neurol..

[31] Rodgers H., Bosomworth H., Krebs H.I., van Wijck F., Howel D., Wilson N., Aird L., Alvarado N., Andole S., Cohen D.L. (2019). Robot assisted training for the upper limb after stroke (RATULS): A multicentre randomised controlled trial. Lancet.

[32] Mauro M., Fasano A., Germanotta M., Cortellini L., Insalaco S., Pavan A., Comanducci A., Guglielmelli E., Aprile I. (2024). Restoring of interhemispheric symmetry in patients with stroke following bilateral or unilateral robot-assisted upper-limb rehabilitation: A pilot randomized controlled trial. IEEE Trans. Neural Syst. Rehabil. Eng..

[33] Nam T.Q., Tien H.V., Van N.A., Dinh Quan N. (2023). Development of an Autonomous Mobile Robot System for Hospital Logistics in Quarantine Zones. The International Conference on Intelligent Systems & Networks.

[34] Tamantini C., di Luzio F.S., Cordella F., Pascarella G., Agro F.E., Zollo L. (2021). A robotic health-care assistant for COVID-19 emergency: A proposed solution for logistics and disinfection in a hospital environment. IEEE Robot. Autom. Mag..

[35] Leoste J., Strömberg-Järvis K., Robal T., Marmor K., Kangur K., Rebane A.M. (2024). Testing scenarios for using telepresence robots in healthcare settings. Comput. Struct. Biotechnol. J..

[36] Yoo H.J., Kim E.H., Lee H. (2024). Mobile robots for isolation-room hospital settings: A scenario-based preliminary study. Comput. Struct. Biotechnol. J..

[37] Laigaard J., Fredskild T.U., Fojecki G.L. (2022). Telepresence robots at the urology and emergency department: A pilot study assessing patients’ and healthcare workers’ satisfaction. Int. J. Telemed. Appl..

[38] Ahn S.Y., Bok S.K., Lee J.Y., Ryoo H.W., Lee H.Y., Park H.J., Oh H.M., Kim T.W. (2024). Benefits of robot-assisted upper-limb rehabilitation from the subacute stage after a stroke of varying severity: A multicenter randomized controlled trial. J. Clin. Med..

[39] Kim J.W., Chen J.T., Hansen P., Shi L.X., Goldenberg A., Schmidgall S., Scheikl P.M., Deguet A., White B.M., Tsai D.R. (2025). SRT-H: A hierarchical framework for autonomous surgery via language-conditioned imitation learning. Sci. Robot..

